# Partial substitution of phosphorus fertilizer with iron-modified biochar improves root morphology and yield of peanut under film mulching

**DOI:** 10.3389/fpls.2024.1459751

**Published:** 2024-10-22

**Authors:** Xiulan Luo, Dewei Wang, Yuting Liu, Yuanze Qiu, Junlin Zheng, Guimin Xia, Ahmed Elbeltagi, Daocai Chi

**Affiliations:** ^1^ College of Water Conservancy, Shenyang Agricultural University, Shenyang, China; ^2^ College of Mechanical and Electrical Engineering, Tarim University, Alar, China; ^3^ Shenyang No.2 High School, Shenyang, China; ^4^ Agricultural Engineering Department, Faculty of Agriculture, Mansoura University, Mansoura, Egypt

**Keywords:** iron-modified biochar, root growth, peanut yield, soil organic matter, water use efficiency

## Abstract

**Introduction:**

Peanut production is being increasingly threatened by water stress with the context of global climate change. Film mulching have been reported to alleviate the adverse impact of drought on peanut. Lower phosphorus use efficiency is another key factor limiting peanut yield. Application of iron-modified and phosphorus-loaded biochar (B_IP_) has been validated to enhance phosphorus utilization efficiency in crops. However, whether combined effect of film mulching and B_IP_ could increase water use efficiency and enhance peanut production through regulating soil properties and root morphologies needs further investigation.

**Methods:**

A two-year (2021-2022) pot experiment using a split-plot design was conducted to investigate the effects of phosphorus fertilizer substitution using B_IP_ on soil properties, root morphology, pod yield, and water use of peanut under film mulching. The main plots were two mulching methods, including no mulching (M0) and film mulching (M1). The subplots were four combined applications of phosphorus fertilizer with B_IP_, including conventional phosphorus fertilizer rates (PCR) without B_IP_, P1C0; 3/4 PCR with 7.5 t ha^-1^ B_IP_, P2C1; 3/4 PCR with 15 t ha^-1^ B_IP_, P2C2; 2/3 PCR with 7.5 t ha^-1^ B_IP_, P3C1; 2/3 PCR with 15 t ha^-1^ B_IP_, P3C2.

**Results and discussion:**

The results indicated that regardless of biochar amendments, compared with M0, M1 increased soil organic matter and root morphology of peanut at different growth stages in both years. In addition, M1 increased peanut yield and water use efficiency (WUE) by 18.8% and 51.6%, respectively, but decreased water consumption by 25.0%, compared to M0 (two-year average). Irrespective of film mulching, P2C1 increased length, surface area, and volume of peanut root at seedling by 16.7%, 17.7%, and 18.6%, at flowering by 6.6%, 19.9%, and 29.5%, at pod setting by 22.9%, 33.8%, and 37.3%, and at pod filling by 48.3%, 9.5%, and 38.2%, respectively (two-year average), increased soil pH and organic matter content during peanut growing season, and increased soil CEC at harvest. In general, the M1P2C1 treatment obtained the optimal root morphology, soil chemical properties, WUE, and peanut yield, which increased peanut yield by 33.2% compared to M0P1C0. In conclusion, the combination of film mulching with 7.5 t ha^-1^ B_IP_ (M1P2C1) effectively improved soil chemical properties, enhanced root morphology of peanut, and ultimately increased peanut yield and WUE.

## 1 Introduction

China is the world’s largest producer and consumer of peanuts, with both total peanut production and consumption accounting for about 40% of the world (Zhang et al., 2020). Due to climate and other planting conditions, peanut planting in China is mostly distributed in Northern region. Taking Liaoning Province as an example, it is characterized by producing high-quality peanut and exporting the majority of peanut in China (Sun et al., 2019). With the increasing planting area of peanuts in Liaoning Province, there are still some issues that limit peanut yield and quality, such as drought and nutrients deficiency. Therefore, it is essential to develop water-saving techniques to ensure peanut production. Plastic film mulching is an efficient method for drought-resistance of crop in farmland. Studies have shown that film mulching could increase soil temperature (Zhao et al., 2023b), reduce soil moisture evaporation, enhance peanut yield (Sun et al., 2018), and ultimately improve WUE of crop (Braunack et al., 2015). Hence, it is important to adopt film mulching in peanuts production to cope with water shortage and increase peanut yield.

Phosphorus, as one of the essential nutrients for peanuts growth, is an important component of nucleic acids, nucleotides, phospholipids, and amino acids (Ajay et al., 2017). Though peanuts prefer phosphorus, due to the lower mobility of phosphorus in soil, it is easy to be fixed and accumulated in the form of phosphate in soil (Gealy, 2015), which are not conducive to the absorption and utilization of phosphorus by peanuts. It is reported that suitable application rate of phosphorus fertilizer can not only optimize root morphology and improve phosphorus absorption and utilization of crop, but also facilitate the formation of rhizobia, increase nitrogen absorption and fixation, and thereby enhance peanuts yield (Bebeley et al., 2024; He et al., 2024). However, excessive application of phosphorus fertilizer will not improve peanut yield or quality, but will strengthen environmental risk induced by serious phosphorus leaching (Taliman et al., 2019; Li et al., 2024). Therefore, seeking efficient phosphorus fertilizer management in farmland is of great importance for improving phosphorus fertilizer utilization efficiency and alleviating eutrophication issues caused by phosphorus leaching.

In recent decades, many efficient phosphorus fertilizer managements have been put forward and developed, such as partial substitution of phosphorus fertilizer with organic fertilizer (Reddy et al., 2000; Liao et al., 2024), combination of microbial agents and phosphorus fertilizer (Zuo, 2005), reduction of phosphorus fertilizer with biochar or biochar-based fertilizer (Dolatmand-Shahri et al., 2024; Qadir et al., 2024), etc. It has been reported that combination of reduced phosphorus fertilizer with optimization measures can effectively improve soil physicochemical properties and phosphorus use efficiency (Xu et al., 2023). Among abovementioned managements, combined application of biochar and phosphorus fertilizer is widely applied in farmland to adsorb phosphorus and reduce phosphorus leaching due to its high adsorption capacity (Cao et al., 2021). However, the physicochemical properties of biochar varied with preparation conditions (Nardis et al., 2022; Řimnáčová et al., 2024; Zhang et al., 2024). Numerous studies have suggested that the adsorption capacity of chemical modified biochar for phosphorus was higher than unmodified biochar (Zheng et al., 2020; Zhao et al., 2022). Among various modification methods, the most commonly used is metal salt modification. After iron salt modification, the surface of biochar will form a certain amount of iron oxide and hydroxyl oxide, thereby enhancing the adsorption of biochar for phosphorus (Yao et al., 2011; Gao and Wan, 2023). Due to the excellent absorption performance of iron-modified biochar for phosphorus, in recent years, scholars proposed a new idea that load iron-modified biochar with phosphorus fertilizer to produce B_IP_, which have been applied in farmland to reduce phosphorus fertilizer application rates (Yuan et al., 2012). Studies have demonstrated that application of B_IP_ could effectively improve phosphorus utilization efficiency (Wu et al., 2022), increase soil available phosphorus, promote phosphorus uptake and utilization in plant, thereby enhancing photosynthesis, dry matter accumulation, and yield of crop (Xia et al., 2023). However, litter information is available about the response of root morphology and pod yield of peanut to B_IP_ application, especially under film mulching. Therefore, further study is needed to explore the potential effect of combined application of B_IP_ and film mulching on peanut production.

In this study, we hypothesized that the application of B_IP_ would improve soil properties, root morphology, pod yield, and water use efficiency of peanut under film mulching. Thus, we aimed to evaluate the effect of B_IP_ on soil organic matter, pH, CEC, root morphology (length, surface area, and volume), peanut yield, water consumption, and WUE under film mulching.

## 2 Materials and methods

### 2.1 Experimental site and materials

**Figure 1:**
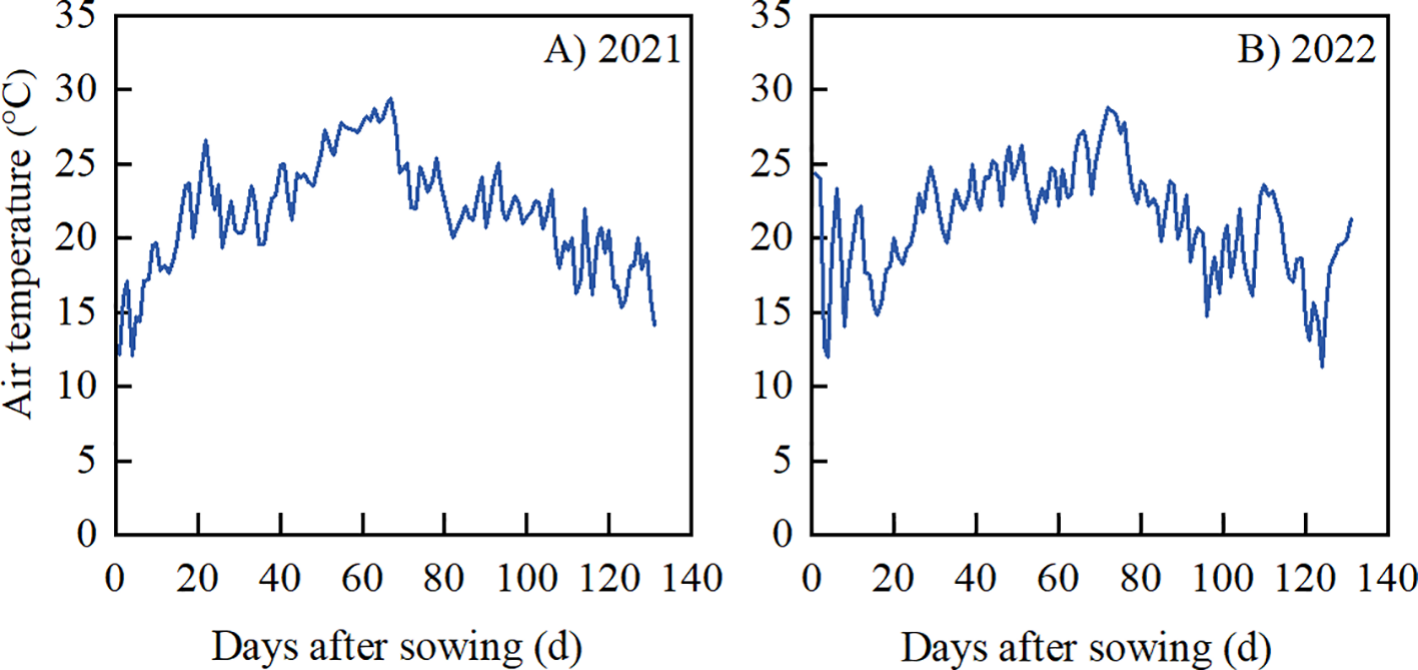
Daily mean air temperature during the peanut growing season in 2021 **(A)** and 2022 **(B)** in Shenyang, Northeast China.

A pot experiment was conducted at the Crop Drought Resistance Cultivation Simulation Test Field of Academy of Agricultural Sciences, Liaoning Province, China (41.53°N, 123.44°E) during peanut growing seasons (May-October) in 2021 and 2022. The soil used for pot experiment was collected locally from the topsoil of 0-30 cm. The physicochemical properties of soil were shown in **Table 1**. The daily average air temperatures during the peanut growing seasons in both years were presented in **Figure 1**.

**Table 1:** Physio-chemical properties of soil used for pot experiment.

Soil texture	Total N (g kg^-1)^	pH	Available P content (mg kg^-1^)	Available K content (mg kg^-1^)	Organic matter content (g kg^-1^)	Soil bulk density (g cm^-3^)	Field capacity (g g^-1^, %)
Silt loam	0.68	6.58	20.16	95.53	8.38	1.38	21.94

The peanut variety used in this study is “Nonghua 9”. The biochar was produced by Shenyang Longtai Biotechnology Co., Ltd., which was pyrolyzed from maize straw at 450° under anaerobic conditions. The biochar had an organic carbon of 515 g kg^-1^, total nitrogen of 10.2 g kg^-1^, total phosphorus of 8.1 g kg^-1^, and total potassium of 15.7 g kg^-1^. The fertilization rates were in line with local farmers’ practices, with 90 kg ha^-1^ N as urea and 90 kg ha^-1^ K_2_O as potassium sulfate. Phosphate fertilizer was applied as superphosphate with 105 kg ha^-1^ P_2_O_5_, as reported by Zheng et al. (2021).

### 2.2 Preparation of B_IP_

Firstly, maize straw-derived biochar and iron containing solution including an equal dose of 1 mol L^-1^ for FeCl_2_ and FeCl_3_ concentrations were prepared in a solid-liquid ratio of 1:15, followed by magnetic stirrer for 30 min. In order to adjust the pH value to 10, a 5 mol L^-1^ NaOH solution was added to the mixture, which was filtered, then dried at 60° and pyrolyzed at 450° for 1 h. Subsequently, the pyrolysis product was passed through a 0.15 mm mesh, washed with deionized water no less than 3 times, and dried at 60° for 48 h.

Afterwards, iron-modified biochar was added to NaH_2_PO_4_ solution containing 200 mg L^-1^ for initial phosphorus concentration in a solid-liquid ratio of 1:200, and then stirred for 12 h. Finally, the iron-modified and phosphorus saturated biochar was washed multiple times with deionized water, and dried to neutrality.

### 2.3 Experimental design

A split-plot experimental design was conducted in both years. The main plots were mulching methods: no film mulching (M0) and film mulching (M1). The subplots were partial substitution of phosphorus fertilizer with B_IP_: conventional phosphorus fertilizer rate (PCR) without biochar amendment, P1C0; 3/4 PCR with 7.5 t ha^-1^ B_IP_, P2C1; 3/4 PCR with 15 t ha^-1^ B_IP_, P2C2; 2/3 PCR with 7.5 t ha^-1^ B_IP_, P3C1; 2/3 PCR with 15 t ha^-1^ B_IP_, P3C2. Each treatment was replicated twelve times. The pots were rewatered to 85% field water capacity (FC) when the soil moisture dropped to 55% FC during the whole growth period (Li, 2018). Field capacity was measured using the overnight free drainage method proposed by de Melo Carvalho et al. (2014) and Suliman et al. (2017). During the experiment, soil moisture was controlled using the gravimetric method (Zheng et al., 2021). Each pot was daily weighed.

The pot was 28 cm in height, with a top and bottom diameter of 32.5 cm and 27 cm, respectively. The bottom of each pot was sealed with the plastic sleeve to avoid water and nutrients losses and facilitate root sampling. Each pot was filled with 12 kg of dry soil, which was passed through a 2 mm sieve, and then watered to 100% FC. Nitrogen, potassium, phosphorus fertilizer, and B_IP_ were all basal applied before sowing. When soil moisture reached about 70% FC, five seeds with uniform size and good fullness were sown in each pot. For the mulching treatment, each pot was fully covered with a black plastic film (0.01 mm thick) with no holes. During the seedling stage, the film was manually punctured to help seedlings emerge. In each pot, seedlings were thinned to two after germination. All pots were placed under an automatic rain shelter to avoid the effect of rainfall on soil moisture. Seeds were sown on 26 May 2021 and 23 May 2022, and harvested on 30 September for both 2021 and 2022.

### 2.4 Sampling and measurements

#### 2.4.1 Soil organic matter, pH, and CEC

From seedling to pod filling, soil samples were collected from 0 to 15 cm depth at three different locations and mixed together for each pot. Soil organic matter was measured using potassium dichromate method (Du and Zhong, 2021). Soil pH was measured using a pH meter (Hangzhou Anheng Instrument Co., Ltd, Hangzhou, Zhejiang Province, CHN) according to Meng et al. (2018). Soil cation exchange capacity (CEC) was determined using the ammonium acetate method.

#### 2.4.2 Root morphology

During each growth stage, three replications for each treatment were selected for root sampling. At the harvest, the shoots of the plant were cut from the roots and the plastic bag was pulled out from the pot and opened to expose the soil and roots. Afterwards, the roots were separated from the soil by gently washing the samples with water. Root samples were then scanned at 400 dpi using an EPSON Expression 110000XL Scanner (Epson America Inc, Long Beach, CA, USA) and analyzed for length, volume, and surface area of root using WinRHIZO LA2400 Software (Regent Instruments Inc, Quebec, QC, CNA).

#### 2.4.3 Peanut yield, water consumption, WUE, and economic benefit

At physiological maturity, peanut yield in each pot was measured independently. Plants were manually threshed and the pods were air dried until attain 14% of the moisture content, then the pod yield was measured. Total water consumption of peanuts during each growing season was calculated according to Zheng et al. (2021) as follows:


(1) $$\text{TWC}=\sum_{i=1}^{n}\left(G_{i}+I-G_{i+1}\right)/\text{S}$$

where TWC is total water consumption (mm), $G_{i}$ and $G_{i+1}$ are pot weight (kg) on the ith day and (i+1)th day (i = 1, 2, 3, 4,…, n), n is the harvest day in the peanut growing season, S is the cross-sectional area of the pot (m^2^), and I is the irrigation water amount each day (kg).

WUE was calculated as follows:


(2) WUE=Y/(10*TWC)

where WUE is water use efficiency (kg m^–3^) and Y is peanut yield (kg ha^–1^).

The economic benefit (EB) of our study excludes the costs of peanut seeds and pest control, mainly from the perspective of major resource inputs and pod yield. The EB was calculated as follows:


(3) $$\text{EB}={\text{C}}_{yield}-{\text{C}}_{fertilizer}-{\text{C}}_{water}-{\text{C}}_{b}-{\text{C}}_{bpp}-{\text{C}}_{pf}$$

where C*_yield_* is the value of pod yield ($1.67 kg^-1^ peanut pod); C*_fertilizer_* is the costs of N, P, and K fertilizers ($0.36 kg^-1^ urea-N; $0.13 kg^-1^ superphosphate; $0.59 kg^-1^ potassium sulfate); C*_water_* is the costs of water usage ($0.028 m^-3^ water usage); C*_b_* is the costs of biochar transportation and application ($109.06 t^-1^); C*_bpp_* is the costs of biochar processing and production ($76.82 t^-1^); C*_pf_* is the cost of plastic film ($0.24 m^-2^).

### 2.5 Statistical analysis

Two years data were respectively analyzed based on the split-plot design with the *Agricolae* package in R studio (Posit PBC Inc, San Francisco, CA, USA) (Mendiburu, 2014). Mulching and B_IP_ as fixed factors and replicates as random factors were considered. Multiple comparisons between treatments were undertaken using Tukey’s HSD test at the 5% probability level. Graphs were produced by the Origin 2021 (OriginLab Corporation Inc, Amherst, MA, USA). Principle component analysis was conducted by the *Factoextra* package in R studio.

## 3 Results

### 3.1 Soil pH, organic matter, and CEC

**Figure 2:**
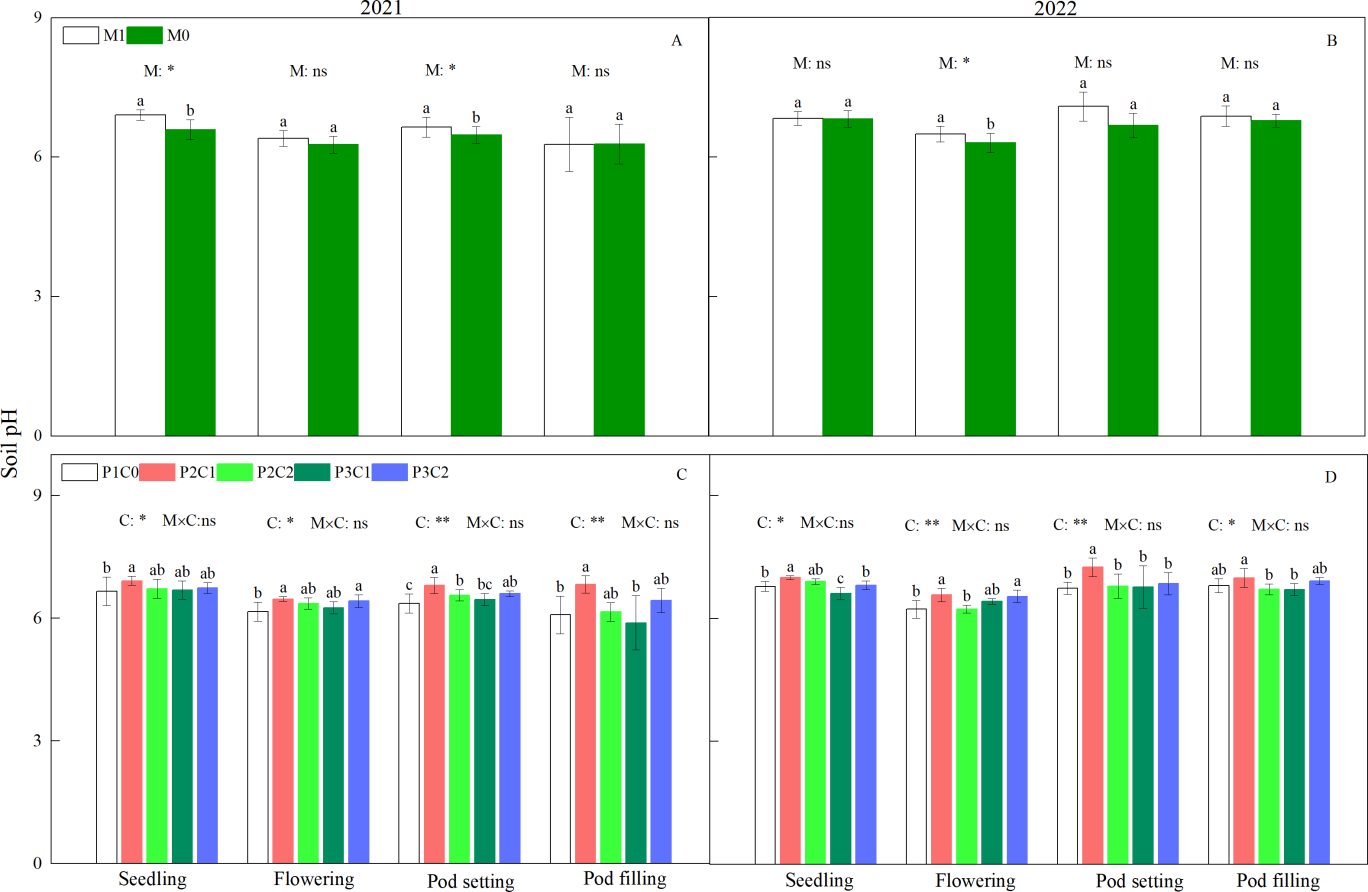
Effect of mulching methods **(A, B)** and biochar amendments **(C, D)** on soil pH during the whole peanut growth stages. M1 and M0 represent film mulching and no mulching control, respectively. P1, P2, and P3 represent conventional phosphorus fertilizer application rate, 3/4 and 2/3 conventional phosphorus fertilizer application rates, respectively. C0, C1, and C2 represent no biochar control, 7.5 t·ha^-1^, and 15 t·ha^-1^ B_IP_, respectively. ** and * indicates significance at 0.01 and 0.05 probability levels, respectively. ns indicates non-significance. For each factor, within each stage, different letters above the bars indicate significant differences between different treatments at *P* < 0.05 level. The same as below.

In 2021, film mulching treatment (M1) had higher (*P* < 0.05) soil pH than the no mulching control (M0) at the seedling stages (**Figure 2A**). In 2021 and 2022, during the whole peanut growth stages, P2C1 significantly increased soil pH compared to the non-biochar control (P1C0). At the flowering stage, compared to P1C0, P2C2 significantly (*P* < 0.05) increased soil pH at pod setting in 2021.

**Figure 3:**
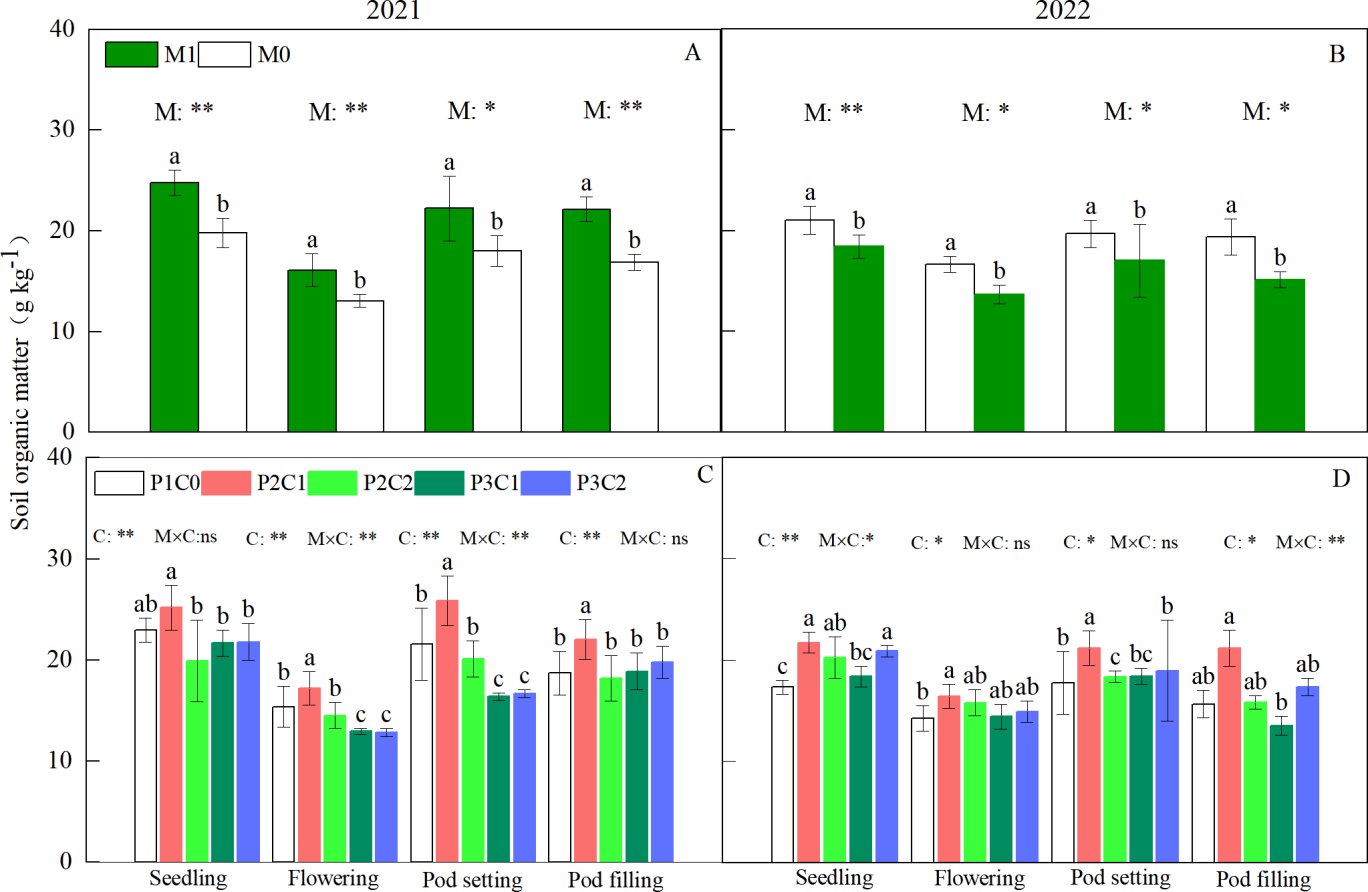
Effect of mulching methods **(A, B)** and biochar amendments **(C, D)** on soil organic matter content during the whole peanut growth stages in 2021 and 2022. For each factor, within each stage, different lowercase letters above the bars indicate significant differences between different treatments at P < 0.05 level. ** and * indicates significance at 0.01 and 0.05 probability levels, respectively. ns indicates non-significance.

**Figure 4:**
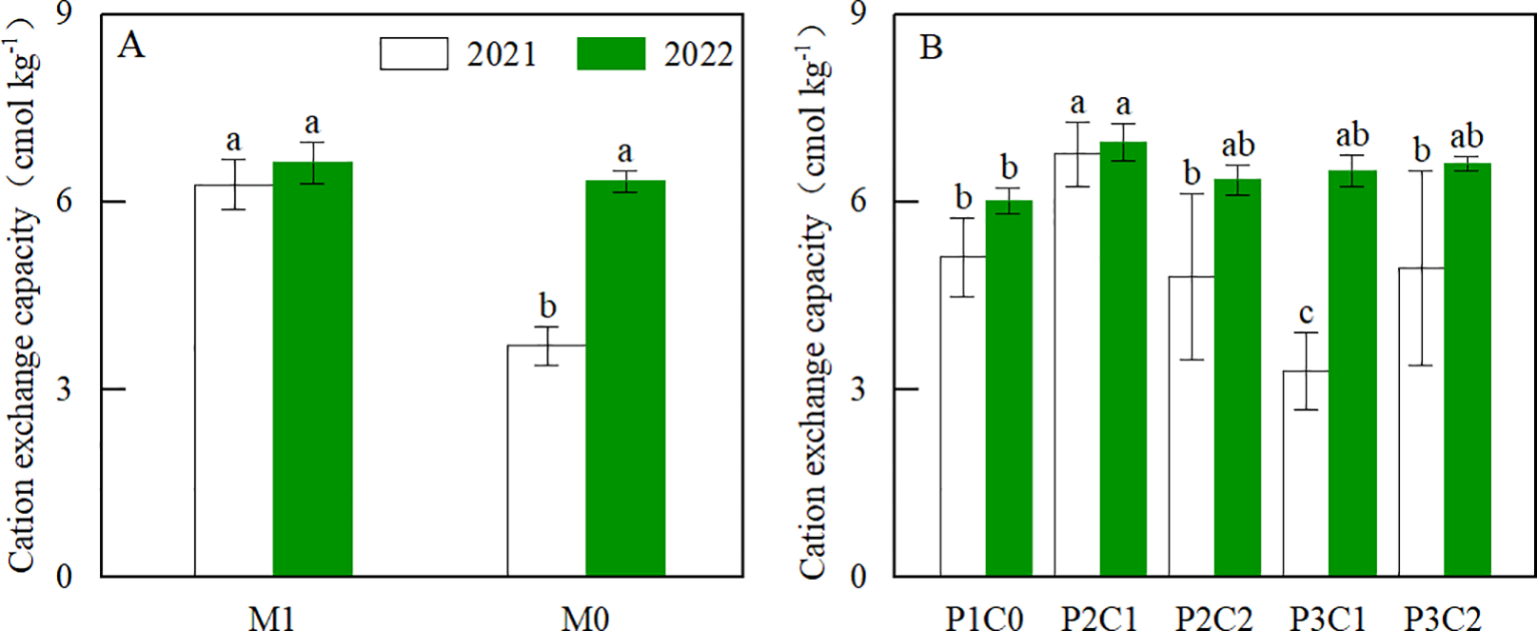
Effect of mulching methods **(A)** and biochar amendments **(B)** on soil CEC at the pod filling stages in 2021 and 2022. For each year, different lowercase letters above the bars indicate significant differences between different treatments at P < 0.05 level.

In both years, M and C treatments had significant (*P* < 0.05) effects on soil organic matter (SOM) during the whole peanut growth stages (**Figure 3**). There was a significant (*P* < 0.05) M × C interaction for SOM at the flowering and pod setting stages in 2021, and at the seedling and pod filling stages in 2022 (**Figures 3C, D**). Compared with M0, M1 increased SOM by 19.8% at the seedling, 22.5% at the flowering, 26.3% at the pod setting, and 22.5% at the pod filling stages (**Figures 3A, B**), respectively (two-year average). Compared with P1C0, P2C1 increased SOM by 24.8% and 36.0% during the flowering and pod setting stages (**Figures 3C, D**), respectively (two-year average). In 2021, M1 had higher (*P* < 0.05) soil CEC than M0 (**Figure 4A**; **Table 2**) at harvest. In both years, P2C1 increased soil CEC by 23.91% (two-year average, **Figure 4B**) compared to the no-biochar control (P1C0).

**Table 2:** The ANOVA for the effect of mulching methods and biochar amendments on soil CEC at the pod filling stage.

Year	Main effects	CEC
2021	M	**
	C	**
	M×C	**
2022	M	ns
	C	**
	M×C	ns

M and C represent mulching methods and biochar amendments, respectively. ** and ns represent extremely significant (*P*<0.01) and non-significance, respectively.

### 3.2 Root morphology

#### 3.2.1 Root length

**Figure 5:**
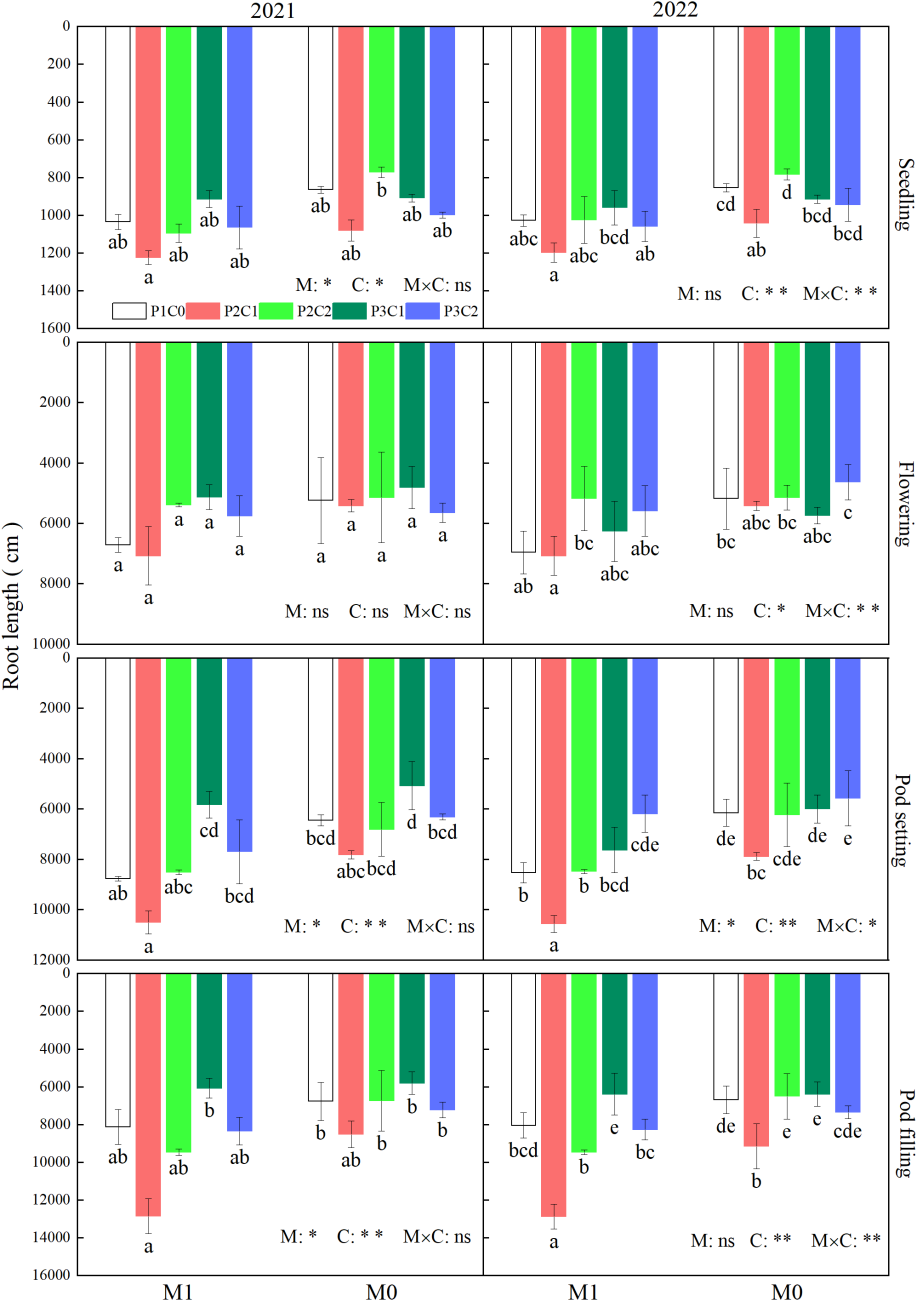
Effects of film mulching and biochar amendments on total root length of peanut during the whole growth stages in 2021 and 2022. For each factor, within each stage, different lowercase letters above the bars indicate significant differences between different treatments at P < 0.05 level. ** and * indicates significance at 0.01 and 0.05 probability levels, respectively. ns indicates non-significance.

As shown in **Figure 5**, mulching methods (M) had a significant (*P* < 0.05) impact on root length at the seedling and pod filling stages in 2021, and at the pod setting stage in both years. Biochar amendments (C) had significant (*P* < 0.05) impacts on total root length during the whole growth stages, except the flowering in 2021. There was significant (*P* < 0.05) interaction of M × C on root length during all growth stages in 2022. Regardless of biochar amendments, compared with M0, M1 increased root length at the seedling, pod filling, and pod setting stages by 12.4%, 19.3%, and 27.2% in 2021, 10.8%, 19.3%, and 30.1% in 2022, respectively. Averaged across film mulching, compared with P1C0, P2C1 increased root length by 20.1% at the seedling, 23.0% at the pod setting, and 46.5% at the pod filling stages, respectively (two-year average).

#### 3.2.2 Root surface area

**Figure 6:**
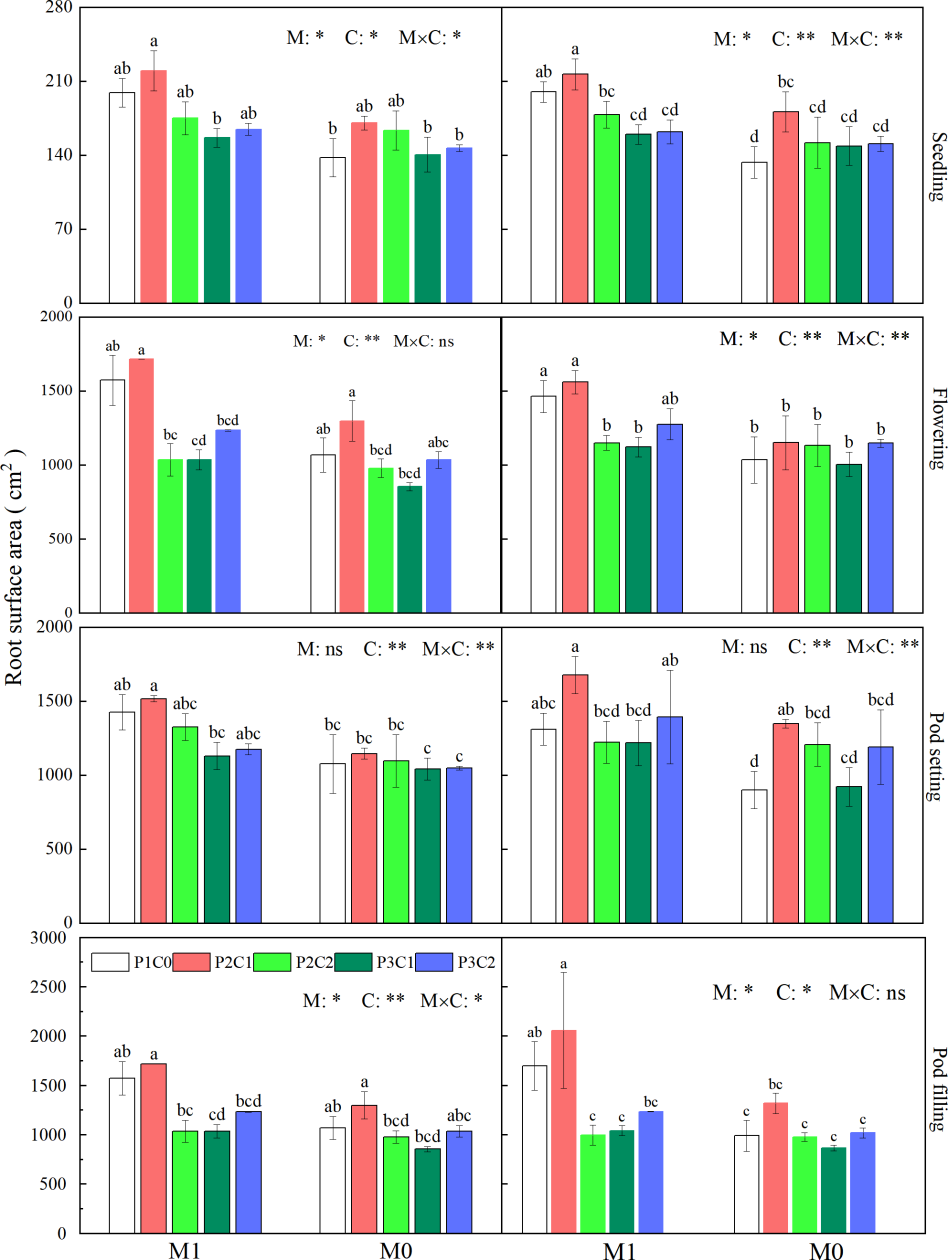
Effects of mulching methods and biochar amendments on root surface area of peanut during the whole growth stages in 2021 and 2022. For each factor, within each stage, different lowercase letters above the bars indicate significant differences between different treatments at P < 0.05 level. ** and * indicates significance at 0.01 and 0.05 probability levels, respectively. ns indicates non-significance.

During the whole growth stages, M1 had significantly (*P* < 0.05) higher root surface area than M0, which was 25.0% to 26.0% higher than M0 except pod setting in both years. The M1P2C1 treatment obtained the highest root surface area among all the treatments, which had similar root surface area with M1P1C0. Moreover, M1P2C1 had higher root surface area than M1P2C2 at flowering and pod filling in 2021, and at all observed stages in 2022. Further, M1P2C1 had higher root surface area than M1P3C1 at all observed stages in both years (**Figure 6**).

#### 3.2.3 Root volume

**Figure 7:**
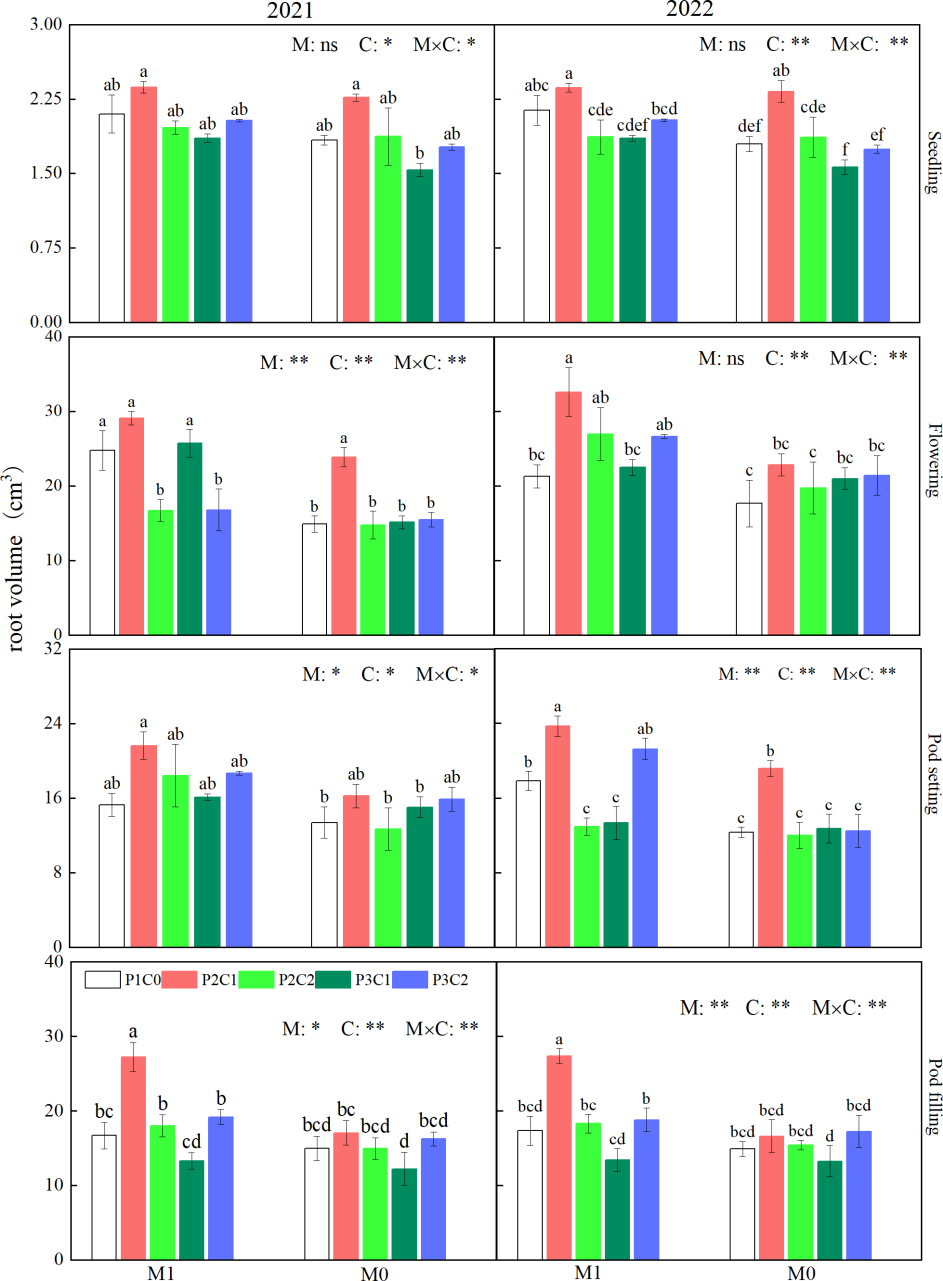
Effects of mulching methods and biochar amendments on root volume of peanut during the whole growth stages in 2021 and 2022. For each factor, within each stage, different lowercase letters above the bars indicate significant differences between different treatments at P < 0.05 level. ** and * indicates significance at 0.01 and 0.05 probability levels, respectively. ns indicates non-significance.

From pod setting to pod filling stages, M1 had significant (*P* < 0.05) higher root volume than M0, which was 24.1% (pod setting), and 13.1% (pod filling) higher than M0, respectively (two-year average). As for biochar amendment, during the whole growth stages, P2C1 increased root volume by 17.9% to 39.8% (2021) and 20.4% to 45.7% (2022), respectively, compared to P1C0 (**Figure 7**). The M1P2C1 treatment had the highest root volume among all the combined treatments during the entire growth stages.

### 3.3 Peanut yield, WUE, and economic benefit

Regardless of biochar amendments, compared with M0, M1 increased (*P* < 0.05) peanut yield by 18.8% (two-year average). Irrespective of film mulching, P2C1 had the highest (*P* < 0.05) peanut yield among all the biochar treatments, which was 14.1% (2022) higher than P1C0 (two-year average). Among all the combined treatments, the M1P2C1 treatment achieved the highest (*P* < 0.05) peanut yield, which was 32.2% (2021) and 34.2% (2022) higher than the M0P1C0 (conventional practice), respectively (**Table 3**).

**Table 3:** Effect of mulching methods and biochar amendments on yield, water consumption, WUE, and economic benefit of peanut.

Treatments	2021				2022			
	Yield (kg ha^-1^)	Total water consumption (mm)	Water use efficiency (kg m^-3)^	Economic benefit ($ ha^-1^)	Yield (kg ha^-1^)	Total water consumption (mm)	Water use efficiency (kg m^-3^)	Economic benefit ($ ha^-1^)
M1	5529.55 ± 417.37a	321.53 ± 13.39b	1.72 ± 0.15a	7474 ± 536a	5848.01 ± 190.59a	208.89 ± 7.72b	2.80 ± 0.24a	6796 ± 445a
M0	4858.87 ± 238.84b	380.75 ± 13.15a	1.28 ± 0.06b	6353 ± 453b	4721.35 ± 63.93b	274.98 ± 9.17a	1.72 ± 0.07b	5807 ± 313a
P1C0	5538.00 ± 247.29a	387.14 ± 19.66a	1.43 ± 0.13b	9030 ± 436a	5055.49 ± 190.59bc	253.51 ± 17.73a	1.99 ± 0.27c	8036 ± 285a
P2C1	5880.58 ± 527.14a	340.59 ± 22.91b	1.73 ± 0.27a	8458 ± 762ab	5796.60 ± 353.44a	234.70 ± 27.14b	2.46 ± 0.55a	7395 ± 720a
P2C2	4920.39 ± 97.71b	341.43 ± 13.63b	1.44 ± 0.08b	5349 ± 277cdP	5156.82 ± 121.83bc	236.63 ± 20.86b	2.18 ± 0.26bc	4863 ± 265c
P3C1	4641.74 ± 144.75b	340.22 ± 24.12b	1.36 ± 0.12b	6846 ± 324bc	4944.51 ± 115.80c	243.26 ± 20.99ab	22.03 ± 0.24c	6168 ± 221b
P3C2	4989.14 ± 301.57b	346.37 ± 20.02b	1.444 ± 0.16b	4885 ± 352d	5494.57 ± 190.59ab	241.69 ± 23.88ab	2.27 ± 0.36b	5045 ± 153c
M1P1C0	5864.90 ± 137.52b	357.83 ± 4.82bcd	1.644 ± 0.04b	9699 ± 662a	5114.60 ± 127.86bc	226.01 ± 1.45b	2.26 ± 0.11c	8445 ± 352a
M1P2C1	6696.02 ± 147.17a	305.97 ± 6.63e	2.19 ± 0.07a	9700 ± 304a	6209.89 ± 85.65a	194.53 ± 3.98c	3.19 ± 0.03a	8884 ± 285a
M1P2C2	4943.31 ± 123.04c	321.53 ± 7.84cde	1.54 ± 0.07bcd	5388 ± 349cd	4776.84 ± 776.00bc	20.527 ± 3.26bc	2.33 ± 0.09bc	5110 ± 441cd
M1P3C1	5032.57 ± 132.69c	304.28 ± 12.06e	1.65 ± 0.04b	6919 ± 684bc	4677.93 ± 83.23cd	212.87 ± 3.62bc	2.20 ± 0.70c	6326 ± 398bc
M1P3C2	5107.36 ± 147.17c	318.39 ± 7.84de	1.60 ± 0.02bc	5663 ± 119bcd	4839.57 ± 100.12b	205.87 ± 10.37bc	2.35 ± 0.12b	5216 ± 240cd
M0P1C0	5063.93 ± 165.26c	416.45 ± 110.73a	1.222 ± 0.05e	8361 ± 256ab	4626.06 ± 8.44d	281.01 ± 55.55a	1.65 ± 0.02d	7628 ± 342ab
M0P2C1	5211.10 ± 91.68bc	375.20 ± 8.20ab	1.39 ± 0.04cde	7216 ± 798abc	4427.02 ± 38.06d	274.98 ± 14.59a	1.61 ± 0.08d	5905 ± 541cd
M0P2C2	4896.26 ± 90.47c	361.45 ± 1.33bc	1.35 ± 0.02cde	5310 ± 511cd	4481.30 ± 97.71cd	267.98 ± 9.65a	1.667 ± 0.08d	4616 ± 308d
M0P3C1	4945.72 ± 120.63c	376.280 ± 2.65ab	1.31 ± 0.06de	6774 ± 232bcd	4488.54 ± 54.28d	273.65 ± 13.03a	1.64 ± 0.10d	6009 ± 245cd
M0P3C2	4174.91 ± 20.51d	374.23 ± 12.54ab	1.12 ± 0.04e	4106 ± 13d	4334.50 ± 15.68cd	277.51 ± 6.63a	1.67 ± 0.04d	4874 ± 173cd
ANOVA								
M	*	**	**	*	**	*	**	ns
C	**	**	**	**	**	*	**	**
M×C	**	ns	**	ns	**	ns	**	**

M1 and M0 represent film mulching and no mulching control, respectively. P1, P2, and P3 represent conventional phosphorus fertilizer application rate, 3/4 and 2/3 conventional phosphorus fertilizer application rates, respectively. C0, C1, and C2 represent no biochar control, 7.5 t·ha^-1^, and 15 t·ha^-1^ iron-modified and phosphorus loaded biochar treatments, respectively. ** and * indicates significance at 0.01 and 0.05 probability levels, respectively. ns indicates non-significance. Means followed by different letters are significantly different at *P* < 0.05 by Tukey’s HSD test.

Irrespective of biochar amendments, M1 reduced (*P* < 0.05) total water consumption by 15.6% in 2021 and 24.0% in 2022, respectively, compared to M0. Regardless of film mulching, P2C1 and P2C2 had less total water consumption than P1C0 in both years. In consideration of the interaction effect, the M1P2C1 treatment had the lowest (*P* < 0.05) total water consumption among all the combined treatments in both years, which was 26.5% and 30.8% lower than M0P1C0, respectively (two-year average).

M1 increased (*P* < 0.05) WUE by 35.4% (2021) and 68.0% (2022), respectively, compared with M0. Irrespective of film mulching, P2C1 achieved the highest WUE among all the biochar treatments, which was 22.9% (2021) and 30.6% (2022) higher than P1C0, respectively. The M1P2C1 treatment obtained the highest (*P* < 0.05) WUE among all the combined treatments in both years.

In view of the M × C interaction, the M1P2C1 treatment obtained the relatively higher economic benefit among all the combined treatments, which was similar with the conventional practice (M0P1C0 treatment) in both years (**Table 3**). From the perspective of biochar treatment, the P2C1 treatment had similar economic benefit with the P1C0 treatment and was significantly higher than other biochar treatments in both years. The P3C2 treatment obtained the lowest economic benefit among all the biochar treatments, which was significantly lower than P1C0 in both years.

### 3.4 Principal components analysis

**Figure 8:**
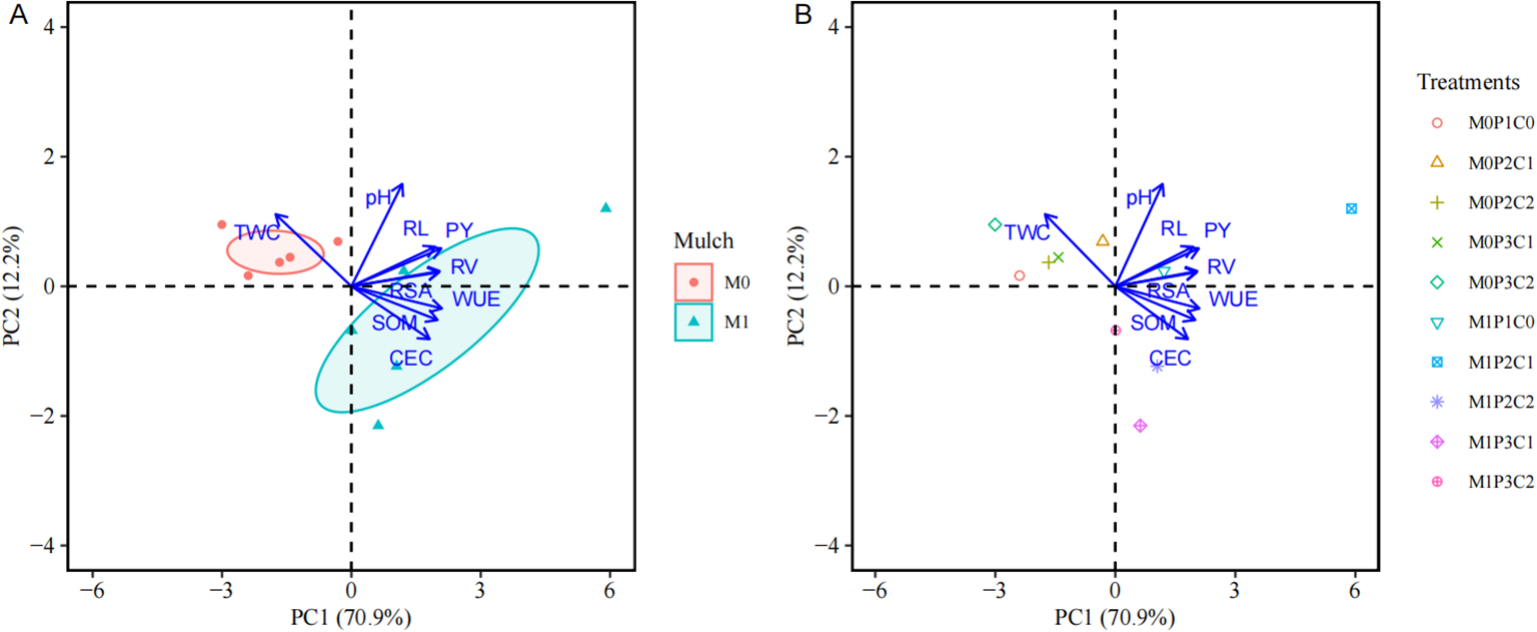
Principal component analysis (PCA) of variables response to mulching methods [**(A)** M1, film mulching; M0, no mulching control] and biochar amendments [**(B)** P1C0, conventional phosphorus fertilizer rate (PCR) without biochar amendment; P2C1, 3/4 PCR with 7.5 t ha^-1^ B_IP_; P2C2, 3/4 PCR with 15 t ha^-1^ B_IP_; P3C1, 2/3 PCR with 7.5 t ha^-1^ B_IP_; P3C2, 2/3 PCR with 15 t ha^-1^ B_IP_] throughout the peanut-growing seasons. The two principal components are plotted with the proportion of variance explained by each component printed next to the axis labels. PY, TWC and WUE represent peanut yield, total water consumption and water use efficiency, respectively; pH, SOM, and CEC represent soil Ph, soil organic matter and cation exchange capacity, respectively; RL, RSA and RV represent root length, root surface area, root volume, respectively.

The PCA results determined two principal components (PCs) with eigenvalues > 1, which explained 83.1% of the total variation in soil chemical properties, root morphology, yield, and water use (**Figure 8**). The first principal component (PC1), accounted for 70.9% of the variability, and mainly contributed to SOM, CEC, root length (RL), root surface area (RSA), root volume (RV), peanut yield (PY), WUE, and total water consumption (TWC). PC2 explained 12.2% of the variability in soil pH.

The correlations between normalized PC1 and PC2 are presented in **Figure 8**. For example, SOM, CEC, and WUE were loaded positively on PC1 but negatively on PC2. TWC were loaded negatively on PC1 but positively on PC2. pH, RL, RSA, RV, and PY were loaded positively on PC1 and PC2. In terms of mulching effect, shifting from M0 to M1 increased all the traits except soil pH, but decreased TWC (**Figure 8A**), indicating that film mulching promoted soil fertility and improved root morphology and peanut yield with less water use. Moreover, among all the combined treatments, the M1P2C1 treatment was closely and positively correlated with SOM, CEC, PY, WUE, RL, RSA, RV, and pH, but negatively correlated with TWC (**Figure 8B**), indicating that partial substitution of phosphorus with iron-modified biochar under film mulching could not only increase peanut yield through improving soil fertility, but also save irrigation water use.

## 4 Discussion

### 4.1 Effects of film mulching and B_IP_ on root morphology of peanut

Compared with other organs, root system of plants was directly in close contact with soil, and its growth was more prone to be affected by soil fertility and moisture. Improved cultivation methods have been developed to improve root morphology, ensuring the absorption of nutrients by plants especially in the middle and later growth stages, promoting dry matter accumulation and yield of crop (Mirsafi et al., 2024). In this study, compared to M0, M1 effectively increased root length (**Figure 5**), root surface area (**Figure 6**), and root volume (**Figure 7**) of peanuts, which was consistent with previous studies (Li et al., 2023; Zhang et al., 2024). These might be due to that film mulching reduced water loss by lowering soil moisture evaporation and raised soil temperature, compared to no mulching control, which provided suitable hydrothermal conditions for roots grow of peanut, thereby improving root morphology and strengthening the transportation capacity of water and nutrients by roots, and ultimately increasing peanut yield (Zhang et al., 2023).

It has been reported that application of porous biochar in soil could optimize root morphology of crop by reducing soil bulk density, increasing soil pores, and improving soil microenvironment (Xia et al., 2022). Similarly, in this study, iron-modified and phosphorus loaded biochar improved root length, root surface area, and root volume of peanuts at various growth stages (**Figures 5**–**7**). This might be resulted from that iron-modified biochar increased soil pores through its larger specific surface area (He et al., 2018), which provided a better space for root growth. Moreover, the increased CEC of iron-modified biochar made it adsorb more nutrients from soil and lately released to supply the growth of peanut roots (Mao et al., 2023), which further optimized root morphology.

Our study also found that the combination of film mulching and 3/4 conventional phosphorus application rate with 7.5 t ha^-1^ B_IP_ obtained the highest root length, root surface area, and root volume among all the treatments, indicating that partial substitution of phosphorus application with appropriate amount iron-modified biochar with film mulching is more conducive to promoting root growth, increasing the contact area between roots and soil, and thereby improving crop water and nutrient absorption through roots, and finally favoring peanut production.

### 4.2 Effects of film mulching and B_IP_ on soil physicochemical properties

Soil is the foundation of agricultural production, and good soil quality is a prerequisite for sustainable development of agriculture (Fan et al., 2022). In this study, the increased soil pH by B_IP_ (**Figure 2**) could be attributed to its alkaline characteristic. When B_IP_ was applied to soil, it reduced the H^+^ content in soil through its adsorption effect, and thereby increasing soil pH (Shaaban et al., 2018). Xu et al. (2019) found that, applying biochar to the soil before sowing slightly increased soil pH compared to no biochar amendment, and soil pH increased with biochar rates. Moreover, the increased soil pH can alter salt exchange performance in soil, enhance the availability of soil nutrients to crop, and ensure better crop growth (Zhou et al., 2024).

Soil organic matter is the main factor determining soil fertility. In this study, both film mulching and B_IP_ increased soil organic matter. Compared with M0, M1 treatment increased soil organic matter during the whole growth stages (**Figure 3**). This might be due to that variations of soil moisture and temperature caused by film mulching affected nutrient transformation in soil, thereby influencing soil organic matter. This is also confirmed by Yang et al. (2023), who found that film mulching increased soil organic matter in corn field by 3.9% compared to no film control in the dam area. Meanwhile, B_IP_ increased soil organic matter compared to no biochar amendment. In this study, P2C1 treatment increased soil organic matter at various growth stages compared to P1C0 (**Figure 3**). This could be attributed to that application of B_IP_ provided abundant nitrogen and carbon sources for the growth of soil microorganisms, which promoted the formation of soil organic matter. In comparison, conventional fertilization only inputs nitrogen sources to the soil, and soil organic matter rapidly decomposes due to the action of microorganisms, leading to the decrease of organic matter in soil. Tian et al. (2016) also demonstrated that biochar could exert the protective effect of soil aggregates on soil organic matter, and enhance the stability of soil organic matter, which ultimately increase soil organic matter content. In addition, compared to no biochar control, B_IP_ increased soil CEC at final harvest over two years (**Figure 4**). This might be resulted from that iron-modified biochar had large surface area and abundant pore structure, which lead to it had strong adsorption capacity for cation and effectively increase soil CEC and fertility (Huang et al., 2022).

### 4.3 Effects of film mulching and B_IP_ on peanut yield, WUE, and economic benefit

In this study, film mulching increased peanut yield compared to no mulching control (**Table 3**), which is similar to Zhao et al. (2023a). This might be because that film mulching increased soil temperature, improved water and nutrients availability to peanut, accelerated peanut growth, increased dry matter accumulation, and ultimately enhanced peanut yield (Sun et al., 2018). Additionally, the WUE of film mulching treatment was higher than that of no mulching control, which could be resulted from that film mulching not only increased peanut yield but also reduced water consumption, as film mulching suppressed gas exchange between soil and atmosphere and reduced the evaporation of ineffective soil moisture, and thus improved WUE (Zhao et al., 2012).

In our study, the application of 3/4 conventional phosphorus application rate with 7.5 t ha^-1^ B_IP_ increased peanut yield by 14.1% (2022), compared with no biochar amendment (**Table 3**). This might be because that B_IP_, which is rich in phosphorus, not only improved soil quality and fertility due to biochar effect, but also compensated the deficiency of nutrients in biochar with loaded phosphorus (Chen et al., 2011). Moreover, B_IP_ slowly released phosphorus to soil to ensure phosphorus supply to crops in the later growth stage of peanut, which would be beneficial for pod filling of peanut and enhance peanut yield. Simultaneously, regardless of film mulching methods, the P2C1 treatment achieved the highest WUE among all the treatments, which was significantly higher than the conventional phosphorus fertilizer treatment (P1C0) (**Table 3**), since iron-modified biochar enhanced peanut yield and reduced water consumption.

The high cost of biochar production limited its widespread application. Sun et al. (2020) reported that high amount of biochar application (20 t ha^-1^ or 40 t ha^-1^) reduced economic benefit by 99.8–229.3% compared with the non-biochar control, due to the high cost of biochar application. However, in our study, the M1P2C1 treatment had similar economic benefit with the conventional practice (M0P1C0 treatment), which was consistent with Bi et al. (2022) (**Table 3**). This might be due to that in the M1P2C1 treatment, the increased benefit of enhanced peanut yield and reduced cost of phosphorus fertilizer by less phosphorus application rate did not offset the expense of biochar production. Although the M1P2C1 treatment did not increase the economic benefit compared with the conventional practice, the reduced phosphorus fertilizer application will be helpful for alleviating environmental risk induced by excess phosphorus leaching, such as eutrophication. Therefore, the application of M1P2C1 treatment is of importance to increase peanut yield and reduce environmental pollution with less phosphorus fertilizer applied. In order to further increase the economic benefit of the M1P2C1 treatment, we will try to reduce the biochar application rate and simplify the preparation processing of B_IP_ in future so as to lower the expense of biochar production.

## 5 Conclusions

Compared with no mulching control, film mulching improved root morphology, and increased peanut yield and WUE with less water consumption, which was due to the reduction of soil evaporation by film mulching and the increase of soil nutrient availability. Iron-modified and phosphorus loaded biochar increased peanut yield and WUE compared to no biochar amendment. Compared with the conventional practice (P1C0), 3/4 conventional phosphorus fertilizer rate with 7.5 t ha^-1^ iron-modified and phosphorus loaded biochar treatment (P2C1) improved root morphology and peanut yield. This could be attributed to that iron-modified biochar increased soil CEC and organic matter content, which promoted soil fertility and made more nutrients available for root absorption and thereby favoring peanut growth. In addition, the enhanced yield could also be linked to increased soil pH since the soil used is acid. In general, application of 3/4 conventional phosphorus application rate with 7.5 t ha^-1^ iron-modified and phosphorus loaded biochar treatment under film mulching (M1P2C1) increased peanut yield and WUE through optimizing root morphology and soil chemical properties with less water consumption.

## Data Availability

The raw data supporting the conclusions of this article will be made available by the authors, without undue reservation.

## References

[B1] Ajay B. C. Meena H. N. Singh A. L. Bera S. K. Dagla M. C. Kumar N.. (2017). Response of different peanut genotypes to reduced phosphorous availability. Indian J. Genet. Plant Breed. 77, 105–111. doi: 10.5958/0975-6906.2017.00014.1

[B2] Bebeley J. F. Kamara A. Y. Jibrin J. M. Tofa A. I. Solomon R. Kamai N. (2024). Effect of combined use of supplementary irrigation, manure and P fertilization on grain yield and profitability of soybean in northern Nigeria. Heliyon 10, 1–11. doi: 10.1016/j.heliyon.2024.e28749 PMC10998218 38586393

[B3] Bi R. Zhang Q. Zhan L. Xu X. Zhang X. Dong Y.. (2022). Biochar and organic substitution improved net ecosystem economic benefit in intensive vegetable production. Biochar 4, 46. doi: 10.1007/s42773-022-00168-9

[B4] Braunack M. V. Johnston D. B. Price J. Gauthier E. (2015). Soil temperature and soil water potential under thin oxo degradable plastic film impact on cotton crop establishment and yield. Field Crops Res. 184, 91–103. doi: 10.1016/jfcr.2015.09.009. J. F. C. R.

[B5] Cao D. Lan Y. Chen W. Yang X. Wang D. Ge S.. (2021). Successive applications of fertilizers blended with biochar in the soil improve the availability of phosphorus and productivity of maize (*Zea mays* L.). Eur. J. Agron. 130, 126344. doi: 10.1016/j.eja.2021.126344

[B6] Chen W. Zhang W. Meng J. Xu Z. (2011). Researches on biochar application technology. Strategic Study CAE. 13, 83–89. doi: 10.3969/j.issn.1009-1742.2011.02.015

[B7] de Melo Carvalho M. T. de Holanda Nunes Maia A. Madari B. E. Bastiaans L. van Oort P. A. J. Heinemann A. B.. (2014). Biochar increases plant-available water in a sandy loam soil under an aerobic rice crop system. Solid Earth 5, 939–952. doi: 10.5194/se-5939-2014

[B8] Dolatmand-Shahri N. Modarres-Sanavy S. A. M. Mirjalili M. H. Mokhtassi-Bidgoli A. (2024). Study the yield and quality of bitter gourd fruit (Momordica charantia) in inoculation with two species of mycorrhizal fungi and phosphorus fertilizer under different irrigation regimes. Plant Physiol. Biochem. 208, 108479. doi: 10.1016/j.plaphy.2024.108479 38461752

[B9] Du M. Zhong H. (2021). Improvement of potassium dichromate oxidation volumetric method for determination of organic matter in soil. Chem. Eng. Management. 25, 16–17. doi: 10.19900/j.cnki.ISSN1008-4800.2021.25.007

[B10] Fan D. De C. Kang Y. Feike A. D. Xue L. Shuang Y.. (2022). Increased soil organic matter after 28 years of nitrogen fertilization only with plastic film mulching is controlled by maize root biomass. Sci. Total Environment. 810, 152244. doi: 10.1016/j.scitotenv.2021.152244 34896135

[B11] Gao A. Wan Y. (2023). Iron modified biochar enables recovery and recycling of phosphorus from wastewater through column filters and flow reactors. Chemosphere 313, 137434. doi: 10.1016/j.chemosphere.2022.137434 36462568

[B12] Gealy D. R. (2015). Deep phosphorus fertilizer placement and reduced irrigation methods for rice (*Oryza sativa* L.) combine to knock-out competition from its nemesis, barnyard grass (Echinochloa crus-galli (L.) P. Beauv). Plant Soil. 391, 427–431. doi: 10.1007/s11104-015-2478-5

[B13] He R. Peng Z. Lyu H. Huang H. Nan Q. Tang J. (2018). Synthesis and characterization of an iron-impregnated biochar for aqueous arsenic removal. Sci. Total Environ. 612, 1177–1186. doi: 10.1016/j.scitotenv.2017.09.016 28892862

[B14] He Z. Dang X. Lin X. Gao G. Liu Y. Ma F. (2024). Combining base to topdressing ratio and layered application of phosphorus fertilizer enhanced cotton yield by regulating root distribution and activity. Soil Tillage Res. 241, 106111. doi: 10.1016/j.still.2024.106111

[B15] Huang K. Kong D. Sheng J. Zhang H. Zu Y. Chen J. (2022). Effect of biochar on physicochemical properties and heavy metal forms of contaminated soil in mining areas. Acta Agriculturae Jiangxi. 34, 73–79. doi: 10.19386/j.cnki.jxnyxb.2022.09.013

[B16] Li Y. (2018). Effect of Yield and Water Use Efficiency Under Biocar-bon Base Fertilizer and Regulated Deficit Irrigation for Peanut (Shenyang: Shenyang Agricultural University).

[B17] Li S. Chen X. Wang Z. Wu D. Wang M. Müeller T.. (2024). Phosphorus fertilizer management for high yields in intensive winter wheat-summer maize rotation system: Integrating phosphorus budget and soil available phosphorus. Field Crops Res. 313, 109410. doi: 10.1016/j.fcr.2024.109410

[B18] Li Z. Wang B. Liu Z. Zhang P. Yang B. Jia Z. (2023). Ridge–furrow planting with film mulching and biochar addition can enhance the spring maize yield and water and nitrogen use efficiency by promoting root growth. Field Crops Res. 303, 109139. doi: 10.1016/j.fcr.2023.109139

[B19] Liao G. Chen X. Yu Y. Chen W. Wang Y. Gao W.. (2024). Effects of phosphorus reduction and organic substitution on yield, quality, and soil fertility of open-field pepper. J. Agro-Environment Science. 43, 617–6265. doi: 10.11654/jaes.2023-0445

[B20] Mao X. Zhai S. Jiang X. Sun J. Yu H. (2023). Effect of modified biochar on physico-chemical properties of farmland soil and immobilization of Pb and Cd and the mechanisms. Environ. Engineering. 41, 113–121+139. doi: 10.13205/j.hjgc.202302016

[B21] Mendiburu FD. (2014). Agricolae: statistical procedures for agricultural research. J. Am. Stat. Assoc. 80. doi: 10.2307/2287932

[B22] Meng J. Tao M. Wang L. Liu X. Xu J. (2018). Changes in heavy metal bioavailability and speciation from a Pb-Zn mining soil amended with biochars from co-pyrolysis of rice straw and swine manure. Sci. Total Environment. 633, 300–307. doi: 10.1016/j.scitotenv.2018.03.199 29574374

[B23] Mirsafi S. M. Sepaskhah A. R. Ahmadi S. H. (2024). Quinoa growth and yield, soil water dynamics, root growth, and water use indicators in response to deficit irrigation and planting methods. J. Agric. Food Res. 15, 100970. doi: 10.1016/j.jafr.2024.100970

[B24] Nardis B. O. Franca J. R. da Silva Carneiro J. S. Soares J. R. Guilherme L. R. G. Silva C. A.. (2022). Production of engineered-biochar under different pyrolysis conditions for phosphorus removal from aqueous solution. Sci. Total Environ. 816, 151559. doi: 10.1016/j.scitotenv.2021.151559 34785233

[B25] Qadir M. F. Naveed M. Khan K. S. Mumtaz T. Raza T. Mohy-Ud-Din W.. (2024). Divergent responses of phosphorus solubilizing bacteria with P-laden biochar for enhancing nutrient recovery, growth, and yield of canola (*Brassica napus* L.). Chemosphere 353, 141565. doi: 10.1016/j.chemosphere.2024.141565 38423145

[B26] Reddy D. D. Rao A. S. Rupa T. R. (2000). Effects of continuous use of cattle manure and fertilizer phosphorus on crop yields and soil organic phosphorus in a Vertisol. Bioresource Technol. 75, 113–118. doi: 10.1016/s0960-8524(00)00050-X

[B27] Řimnáčová D. Bičáková O. Moško J. Straka P. Čimová N. (2024). The effect of carbonization temperature on textural properties of sewage sludge-derived biochars as potential adsorbents. J. Environ. Manage. 359, 120947. doi: 10.1016/j.jenvman.2024.120947 38718599

[B28] Shaaban M. Van Z. L. Bashir S. Younas A. Nunez-Delgado A. Chhajro M. A.. (2018). A concise review of biochar application to agricultural soils to improve soil conditions and fight pollution. J. Environ. Management. 228, 429–440. doi: 10.1016/j.jenvman.2018.09.006 30243078

[B29] Suliman W. Harsh J. B. Abu-Lail N. I. Fortuna A. M. Dallmeyer I. Garcia-Pérez M. (2017). The role of biochar porosity and surface functionality in augmenting hydrologic properties of a sandy soil. Sci. Total Environ. 574, 139–147. doi: 10.1016/j.scitotenv.2016.09.025 27627689

[B30] Sun L. Deng J. Fan C. Li J. Liu Y. (2020). Combined effects of nitrogen fertilizer and biochar on greenhouse gas emissions and net ecosystem economic budget from a coastal saline rice field in Southeastern China. Environ. Sci. pollut. Res. 27, 17013–17022. doi: 10.1007/s11356-020-08204-6 32146660

[B31] Sun T. Li G. Ning T. Zhang Z. Mi Q. Lal R. (2018). Suitability of mulching with biodegradable film to moderate soil temperature and moisture and to increase photosynthesis and yield in peanut. Agric. Water Manage. 208, 214–223. doi: 10.1016/j.agwat.2018.06.027

[B32] Sun H. Yu H. Yu S. Shi P. Ren L. Zhang X. (2019). Current situation and development suggestions of peanut industry in Liaoning Province. Bull. Agric. Sci. Technology. 11), 57–59.

[B33] Taliman N. A. Dong Q. Echigo K. Raboy V. Saneoka H. (2019). Effect of phosphorus fertilization on the growth, photosynthesis, nitrogen fixation, mineral accumulation, seed yield, and sed quality of a soybean low-phytate line. Plants. 8, 119. doi: 10.3390/plants8050119 31071932 PMC6572685

[B34] Tian J. Wang J. Dippold M. Gao Y. Blagodatskaya E. Kuzyakov Y. (2016). Biochar affects soil organic matter cycling and microbial functions but does not alter microbial community structure in a paddy soil. Sci. Total Environ. 556, 89–97. doi: 10.1016/j.scitotenv.2016.03.010 26974565

[B35] Wu H. Zheng Q. Zhang S. Cheng Y. Wang X. (2022). Effect of combined application of magnesium modified biochar and phosphorus fertilizer on phosphorus availability and wheat yield in red soil. Soil Fertilizer Sci. China. 299, 84–90. doi: 10.11838/sfsc.1673-6257.21457

[B36] Xia G. Liu Y. Luo X. Pang Y. Zheng J. Chi D. (2023). Effects of iron-modified and phosphorus loaded biochar on the phosphorus utilization and yield of peanut under film mulching. Trans. Chin. Soc. Agric. Engineering. 39, 176–187. doi: 10.11975/j.issn.1002-6819.202308212

[B37] Xia G. Wang Y. Wang S. Yang Q. Chi D. (2022). Effects of irrigation Methods and biochar on Peanut Root, Phosphorus Utilization and Yield. Trans. Chin. Soc. Agric. Machinery. 53, 316–326. doi: 10.6041/j.issn.1000-1298.2022.02.034

[B38] Xu Y. Wang S. Li J. Hua D. Guo Z. (2019). “Effects of applying swine and fruit biochar on soil nutrient changes,” in Proceedings of the 2019 National Academic Annual Conference on Environmental Engineering (Volume 2) (Beijing, China: Industrial Architecture Magazine Co., Ltd), Vol. 2019. 966–970.

[B39] Xu M. Wang Y. Nie C. Song G. Xin S. Lu Y.. (2023). Identifying the critical phosphorus balance for optimizing phosphorus input and regulating soil phosphorus effectiveness in a typical winter wheat–summer maize rotation system in North China. J. Integr. Agric. 22, 3769–3782. doi: 10.1016/j.jia.2023.05.030

[B40] Yang W. Dai J. Liu Z. Deng X. Yang Y. Zeng Q. (2023). Film mulching alters soil properties and increases Cd uptake in Sedum alfredii Hance-oil crop rotation systems. Environ. pollut. 318, 120948. doi: 10.1016/j.envpol.2022.120948 36574807

[B41] Yao Y. Gao B. Mandu I. Andrew R. Z. Cao X. Pratap P.. (2011). Biochar derived from anaerobically digested sugar beet tailings: Characterization and phosphate removal potential. Bioresource Technol. 102, 6273–6278. doi: 10.1016/j.biortech.2011.03.006 21450461

[B42] Yuan Y. Tian Y. Zhao L. Meng H. (2012). Research progress in the application of biochar. Renewable Energy Resources. 30, 45–49. doi: CNKI:SUN:NCNY.0.2012-09-014

[B43] Zhang H. Feng X. Wang L. Li G. Qiao L. Wei D.. (2020). Establishing a peanut big data plat form in China: A proposal and applications. J. Agric. Big Data. 2, 45–52. doi: CNKI:SUN:NYDS.0.2020-01-008

[B44] Zhang J. Liu X. Wu Q. Qiu Y. Chi D. Xia G.. (2023). Mulched drip irrigation and maize straw biochar increase peanut yield by regulating soil nitrogen, photosynthesis and root in arid regions. Agric. Water Manage. 289, 108565. doi: 10.1016/j.agwat.2023.108565

[B45] Zhang S. Wei L. Trakal L. Wang S. Shaheen S. M. Rinklebe J.. (2024). Pyrolytic and hydrothermal carbonization affect the transformation of phosphorus fractions in the biochar and hydrochar derived from organic materials: A meta-analysis study. Sci. Total Environ. 906, 167418. doi: 10.1016/j.scitotenv.2023.167418 37774876

[B46] Zhao J. Liu Z. Lai H. Zhao M. Zhu Q. Zhao C.. (2023a). The impacts of soil tillage combined with plastic film management practices on soil quality, carbon footprint, and peanut yield. Eur. J. Agron. 148, 126881. doi: 10.1016/j.eja.2023.126881

[B47] Zhao X. Qin X. Li T. Cao H. Xie Y. (2023b). Effects of planting patterns plastic film mulching on soil temperature, moisture, functional bacteria and yield of winter wheat in the Loess Plateau of China. J. Integr. Agric. 22, 1560–1573. doi: 10.1016/j.jia.2023.02.026

[B48] Zhao D. Qiu S. Li M. Luo Y. Zhang L. Feng M.. (2022). Modified biochar improves the storage capacity and adsorption affinity of organic phosphorus in soil. Environ. Res. 205, 112455. doi: 10.1016/j.envres.2021.112455 34863688

[B49] Zhao H. Xiong Y. Li F. Wang R. Qiang S. Yao T.. (2012). Plastic film mulch for half growing-season maximized WUE and yield of potato via moisture-temperature improvement in a semi-arid agroecosystem. Agric. Water Manage. 104, 68–78. doi: 10.1016/j.agwat.2011.11.0167

[B50] Zheng J. Wang S. Wang R. Chen Y. Kadambot H. M. S. Xia G.. (2021). Ameliorative roles of biochar-based fertilizer on morpho-physiological traits, nutrient uptake and yield in peanut (*Arachis hypogaea* L.) under water stress. Agric. Water Management. 257, 107129. doi: 10.1016/j.agwat.2021.107129

[B51] Zheng Q. Yang L. Song D. Zhang S. Wu H. Li S.. (2020). High adsorption capacity of Mg–Al-modified biochar for phosphate and its potential for phosphate interception in soil. Chemosphere 259, 127469. doi: 10.1016/j.chemosphere.2020.127469 32640377

[B52] Zhou J. Tang S. Pan W. Liu X. Han K. Si L.. (2024). Long-term non-flooded cultivation with straw return maintains rice yield by increasing soil pH and soil quality in acidic soil. Eur. J. Agron. 159, 127208. doi: 10.1016/j.eja.2024.127208

[B53] Zuo L. (2005). Effects of microbial inoculum applied with different fertilizers on soil fertility and corn growth. Shanxi Agric. Univ. 3, 25–27.

